# “I hope to get some hope”: balancing expectations, hope, and acceptance in naturally occurring consultations without a medical diagnosis

**DOI:** 10.1080/17482631.2025.2554486

**Published:** 2025-09-02

**Authors:** Trine C. B. Andersen, Sarah Nettleton, Åge Wifstad, Olaug S. Lian

**Affiliations:** a Department of Community Medicine, UiT The Arctic University of Norway, Tromsø, Norway; b Department of Sociology, University of York, York, UK

**Keywords:** Uncertainty, chronic pain, clinician-patient communication, illness experience, clinical interaction

## Abstract

**Background:**

Chronic musculoskeletal pain (CMP) is a prevalent condition often lacking clear biomedical explanations, leading to frustration for both clinicians and patients. The uncertainty surrounding CMP deeply affects the dynamics of clinical consultations.

**Aim:**

This study investigates how clinicians and patients navigate expectations and uncertainty in CMP consultations, focusing on the interplay between hope and acceptance.

**Methods:**

Nineteen naturally occurring consultations were observed and post-consultation interviews conducted with both patients and clinicians. Data were analysed using reflexive thematic analysis and presented through four individual consultations.

**Results:**

The study identified three main themes: expectations, hope, and acceptance. Patients frequently expressed hope for a diagnosis and treatment, while clinicians sought to guide them towards acceptance of their condition and managing symptoms rather than seeking a cure. However, patients often struggled with this approach, viewing it as a loss of hope for improvement. Additionally, patients’ cultural expectations of biomedical solutions affected their response to acceptance-focused communication. The study highlights the tension between encouraging acceptance and maintaining hope.

**Conclusion:**

The findings underscore the need for more nuanced communication strategies that strikes the right balance between fostering hope and acceptance, while acknowledging the cultural expectations of biomedical solutions that patients bring into the consultation room.

## Introduction

Uncertainty is a prominent aspect of healthcare, as all clinical encounters deal with something unknown (Mackintosh & Armstrong, 2020). This is also true for the subject of this paper: consultations between clinicians and patients with chronic musculoskeletal pain (CMP). CMP is defined as pain in bones, joints, or tissues lasting longer than three months (Booth et al., 2017, p. 413). CMP is experienced by 20–33% of the world’s population (WHO, 2022) and common forms include low back pain, neck pain and rheumatoid arthritis. A clear biomedical cause is often absent, particularly for low back and neck pain, leaving clinicians and patients searching for answers and solutions (Hartvigsen et al., 2018). CMP is also often comorbid with mental illness, such as depression and anxiety, although the temporal relationship remains to be determined (Bondesson et al., 2018).

This paper draws on nineteen naturally occurring consultations in a specialized outpatient clinic that uses a biopsychosocial framework and aspects of cognitive behavioural therapy (Buchbinder et al., 2020; Engel, 1977; Miaskowski et al., 2020). This approach addresses both physical and psychological aspects of chronic pain, giving clinicians time to explore patients’ experiences while focusing on reassurance and encouraging an active life (T. C. B. Andersen et al., 2024). Treatment often include physiotherapy, physical activity, adjusted work schedules, or referrals to pain management retreats.

While previous studies have explored communication strategies and expectation management in chronic pain consultations (Wiering et al., 2018), we know less about how patients and clinicians jointly navigate uncertainty, expectations, and therapeutic optimism in real-time. We therefore investigate how these dynamics unfold, particularly how patients and clinicians navigate between expectations, hope, and acceptance when faced with uncertainty.

### Uncertainty in clinician encounters

Uncertainty in clinical consultations may be both medical and existential (Lian et al., 2021). Medical uncertainty relates to diagnostic, aetiological and prognostic limitations (Bhise et al., 2017; Helou et al., 2020). Existential uncertainty, by contrast, concerns how patients experience illness in relation to their identity, and our awareness of our future as open and undetermined (Adamson, 1997).

When a medical diagnosis is lacking, uncertainty increases, and interactions become more complex (Bhise et al., 2017; Lian et al., 2021; Nettleton, 2006). Diagnosis traditionally provides structure and legitimacy (Jutel, 2009). Patients with unexplained chronic conditions might experience disbelief, stigmatization, and marginalization, particularly when symptoms cannot be verified through technology (Edwards et al., 2010; Lian & Robson, 2017; Lillrank, 2003; Nettleton, 2006). In this way, uncertainty can significantly influence the interactional dynamics of a medical consultation.

### Expectations, hope, and acceptance amid uncertainty

Patients and clinicians bring expectations to the clinical encounter. These expectations, defined as “a strong belief that something will happen or be the case” (Stevenson, 2010), can shape how consultations unfold. Patients often anticipate diagnosis, causal explanations, or treatment plans (Geurts et al., 2017; Kee et al., 2023; Verbeek et al., 2004), and may expect more than clinicians can provide or even recommend (Kee et al., 2023; Verbeek et al., 2004).

Researchers distinguish between “predicted” expectation (what is likely) and “ideal” expectations (what is desired), the later often expressed as hope (Leung et al., 2009; Thompson & Suñol, 1995). Patients can thus at the same time hope that clinicians will give them a diagnosis, while expecting to be told that nothing is wrong. While some define hope as a type of expectation others treat them as two independent constructs (Kube, Blease, et al., 2019; Leung et al., 2009). We follow the latter view.

Within a health care setting, hope has been defined as “a confident yet uncertain expectation of achieving a future good which, to the hoping person, is realistically possible and personally significant” (Dufault & Martocchio, 1985, p. 380). What here separates hope and expectation is the uncertainty linked to hope (Kube, Blease, et al., 2019; Kube, Rief, et al., 2019; Leung et al., 2009). While expectations are based on *assumptions* about what will happen in the future (which involves a high probability), hope is a *wish* for something to happen, regardless of likelihood and what one’s expectations might be.

Within a medical context it is in the clinician’s power to create, modify, or even destroy patients’ hope (Eliott & Olver, 2002). However, it is in the clinician’s interest to sustain some amount of hope, as a high level of hope has been linked to positive outcomes such as improved survival, better emotional regulation, and lower levels of depression and anxiety (Corn et al., 2022; Feldman & Snyder, 2005; Hassija et al., 2012). Literature on chronic pain shows that patients often sustain hope, despite limited prospects and moments of despair (Corbett et al., 2007; Eaves et al., 2016). In these cases, a balance between hope and realism is key (Campbell et al., 2010; Clayton et al., 2008; Kube, Blease, et al., 2019; Lannie & Peelo-Kilroe, 2019).

One way for clinicians to modify hope is through acceptance. Pain acceptance implies “a willingness to experience continuing pain without needing to reduce, avoid, or otherwise change it” (McCracken, 1999, p. 93). Pain acceptance has been linked to better adjustment, higher quality of life, better day-to-day functioning, and decreased symptoms and disability (Aymerich et al., 2022; Kratz et al., 2007; McCracken, 1998; Tangen et al., 2020; Vowles et al., 2007). Acceptance is often seen as a sign that patients are ready to cope and self-manage the condition (Samwiri Nkambule & Msiska, 2024). For some, this comes naturally as hope fades; others resist acceptance, especially when the condition is unpredictable and uncertain (Lieberwerth & Niemeijer, 2024). The clinical challenge thus lies in sustaining patients’ hope, while also providing realistic information and promoting acceptance.

## Material and methods

Data collection took place between January and March 2022 at a specialist outpatient rehabilitation clinic in a regional Norwegian hospital. Here we observed and audio-recorded 19 naturally occurring consultations between clinicians and patients presenting with CMP or fatigue. Immediately after each consultation, we conducted individual semi-structured interviews with both patients and clinicians to explore their respective experiences.

### Participants and recruitment

Consultations involved 7 clinicians (2 doctors, 1 occupational therapist and 4 physiotherapists) and 19 patients with CMP (,12 women and 7 men). Eligible patients were aged 18 and 60, fluent in Norwegian, and referred for severe chronic pain or fatigue. Those with severe learning disabilities or limited language skills were excluded. While we strived for an equal gender distribution, the small majority of women reflects the typical patient group (Bjørneboe et al., 2024).

All eligible patients within the data collection period received written information about the study prior to their scheduled appointment. Before the consultation, the study was reintroduced, and written informed consent was obtained (see Table 1 for an overview of all participants). The project was also presented to the clinical staff and 8 of 11 clinicians agreed to participate. Due to anonymity concerns, we do not describe individual clinician characteristics, but interactions consistently reflected efforts to reassure patients about their condition (T. C. B. Andersen et al., 2024). While interactions were generally warm and supportive, shared understanding of the pain was not always achieved (T. C. B. Andersen & Lian, 2025).

**Table 1.** Data overview.

Consultation ID	Clinician ID	Duration (min)	Patient ID	Patient age	Patient gender	Pain Area	Interview P (min)	Interview C (min)
C01	C1	92	P1	52	Man	Shoulder	23	29
C02	C2	109	P2	33	Woman	Neck and back	50	48
C03	C3	75	P3	59	Man	Back and shoulder	25	38
C04	C1	64	P4	51	Woman	Back	33	18
C05	C4	108	P5	30	Man	Back	37	43
C06	C1/C5/C2	83	P6	35	Woman	ME	32	15
C07	C1	69	P7	59	Woman	Hip	22	12
C08	C1	81	P8	29	Woman	Back, neck and head	29	15
C09	C6	130	P9	52	Man	Back, neck and hip	23	41
C10	C1/C3/C7	140	P10	36	Woman	Pelvis and wrists	23	22
C11	C1/C5	85	P11	29	Woman	ME	25	18
C13	C3	93	P13	52	Man	Neck	23	23
C15	C4	30	P15	55	Woman	Numbness in hands	12	13
C16	C2	70	P16	26	Woman	Hypermobility/pain	15	17
C17	C4	63	P17	58	Man	Lower back to foot	53	20
C18	C8	100	P18	30	Woman	Shoulder and lower back	21	37
C19	C8	142	P19	20	Woman	Lower back	13	24
C20	C6 + C7	91	P20	47	Woman	Right hip	29	18
C21	C4	56	P21	49	Man	Lower back and foot	22	19
C22	C2	74	P22	21	Woman	Hypermobility/pain	22	26
C23	C3	54	P23	54	Woman	Lower back to foot	32	16
Average		86		42			27	24

All interviews were carried out the same day, except in C09 where the interview with C3 took place the following day. C12 and C14 are not included in the dataset. C06 and C11 each had three separate consultations with different clinicians. The duration indicates the average for the three consultations and interviews

As part of standard practice, referred patients completed a questionnaire assessing symptoms and impact on daily life. This included sub-measures such as the Hospital Anxiety and Depression Scale (HADS), the Pain Catastrophizing Scale (PCS), and the Pain Self-Efficacy Questionnaire (PSEQ). These measures can provide indicators of psychological comorbidities, including anxiety and depression. While HADS scores did not suggest probable mood disorders in our patient group, no formal diagnostic data were collected. Underlying mental health, if present, could have influenced the dynamics observed; this is acknowledged as a limitation.

### Data collection

All observations and interviews were facilitated by TA who has a background in social anthropology. During consultations, TA was seated in a corner with partial or full view of both participants and did not interact. The clinician sat at a desk with the patient to the side, allowing eye contact, note-taking, and examination without the desk acting as a barrier. In addition to audio-recordings, TA also took field notes capturing atmosphere, non-verbal behaviours, and contextual observations. These notes were later used in interpreting the dialogues and without them important contextual nuances would have been missed.

Interviews were conducted on site in clinic rooms. Before each interview, participants were again given the opportunity to ask questions about the study and were encouraged to share both positive and negative experiences. The semi-structured interview-guide covered themes such as expectations; information/explanations; shared decision making; choice; trust; understanding; future; satisfaction. Patient interviews lasted between 13 and 53 minutes (27 minutes average) and clinician interviews lasted 12 to 48 minutes (24 minutes average). After each set of consultation and interviews, TA took field notes which documented the atmosphere, the main topics discussed, observations and personal reflections. Audio-recordings were transcribed verbatim and fully anonymized.

### Ethical considerations

All participants provided informed written consent prior to enrolment in the study and all received written information about the purpose of the study. The study was approved by the Norwegian Centre for Research Data approval no. no. 807617). Participants were assured about the confidentiality of their information and the option to withdraw consent at any time without compromising their medical care.

### Data analysis

To explore patterns observed across consultations, the data were analysed using tools from reflexive thematic analysis (RTA) (Braun & Clarke, 2022, p. 4). The data were coded by TA in NVivo based on a codebook deriving from coding the first five cases, agreed upon by TA and OSL. Coding process was inductive and data-driven, though some preconceived themes appeared. The research team includes researchers from sociology, anthropology, and philosophy, who have all previously worked with uncertainty in medical settings. In the interpretation, the team reflected on their emergent understandings and embodied experiences with uncertainty in clinical consultations.

Themes such as “expectations”, “hope” and “acceptance” emerged naturally from the data. To ensure the trustworthiness, we followed criteria for qualitative research integrity, including credibility, dependability, transferability, and confirmability (Nowell et al., 2017). Credibility was strengthened through triangulation of data; observation, interviews, and field notes. Dependability and confirmability were reinforced through transparent coding and team-based discussions. Transferability was supported by rich case descriptions.

During analysis, TA and OSL reviewed consultations individually and then collectively, ensuring thematic coherence. Through this iterative process and group discussion, the themes were refined and connected to broader theoretical concepts. “Hope” and “acceptance” became interrelated sub-themes under the overarching theme of “Navigating expectations and uncertainty in chronic musculoskeletal pain consultation”. The final thematic structure is presented in Table 2.

**Table 2.** Thematic structure.

Overarching theme	Sub-theme	Description	Sub-theme/code	Example quote
Navigating expectations and uncertainty in chronic musculoskeletal pain consultation	Hoping instead of expecting	Expressions of desire for a certain thing to happen despite low probability	Hoping for hope	”(…)I hope to get some hope maybe.”
			Hoping for a quick fix	“(…) I had hoped that uh they would be able to say that uh ‘then we have to try to do such and such because the cause is this ligament here’.”
			Hoping for a diagnosis	“I had almost hoped that I would have such Bekhterev’s [ankylosing spondylitis] when I got arthritis.”
			Hoping for surgery	“When I was with the GP last time and she referred me, I might have had a little hope of surgery.”
	Acceptance of uncertainty and a future with pain	Acknowledgement of living with pain and limitations.	Accepting pain and limitations	“So if you can’t do everything at the moment then maybe you simply have to cut back a little. ((pause 2 sec)) and acc- try to accept (.) now you have rheumatism and you have to take care of it.”
			Rejecting acceptance of pain and limitations	“Yes, a bit like ‘isn’t there something more we can do’.”

To deepen our analysis, we selected four illustrative consultations (see Table 3) that offered particularly rich examples of how expectations, hope, and acceptance manifest within the unique dynamics of each interaction. The cases were selected because they reflect key patterns across the dataset and offer theoretical depth. Attention was particularly paid to how each consultation unfolded, and the integrity and context of the interaction was respected by “quoting long extracts and analysing components in light of the whole” (Lian et al., 2024, p. 780). In the following presentation of data, we use “‘(…)’” to indicate an omission for anonymization or brevity reasons and we use “‘[…]’” to indicate an addition of information by the authors.

**Table 3.** Overview of case participants.

Patient	Age	Gender	Work Status	Pain Profile	Background Summary
Freja	Late 20s	Female	50%	~1.5 years back, neck, and head pain; ~6 months new specific back pain.	Accident ~1.5 years ago; Postponed dream education due to accident and pain; Does physiotherapy, yoga and meditation; Hopes for hope.
Jakob	Late 50s	Male	100%	1+ years leg and shoulder pain.	Stroke 3+ years ago; Has tried every type of treatment; Hopes for quick fix.
Anne	Early 30s	Female	100%	~6 months back and shoulder pain (post-RA treatment).	Recent RA diagnosis; mistrust stemming from past clinical encounters; Has tried physiotherapy; Hopes for diagnosis.
Emil	Early 50s	Male	0%	~6–9 months neck, hip, and back pain.	Accident ~1 year ago; Has tried every type of treatment; Evaluated but rejected for surgery. Hopes for surgery

Note: All identifying details have been removed or generalized to maintain participant confidentiality.

We take an interactionist and structural perspective, meaning that we seek to capture individual level interactions as they occur, and locate them in their socio-cultural context. While our empirical exploration focuses on in situ consultations between patients and clinicians as they unfold, we interpret it as interaction between two parties representing different institutionalized positions of patient and clinician (Bourdieu, 1989; Freidson, 1970; Strong, 1979). The interaction is mediated by their different and asymmetrical positions in the social system. Asymmetrical because clinicians have professional authority, which rests on their formal training and clinical competence and the belief that they will use their skills in a beneficial way for the patient (Carel & Kidd, 2017; Parsons, 1951). As such we see the consultation room as culturally created and in this, clinicians and patients are not just individuals but also two roles that meet in a culturally defined system.

## Results

In this result section we will show the complexity of interactions in clinical consultations, where uncertainty dominates, and how the concepts of hope and acceptance unfold and overlap during four separate consultations. Though this diverges from conventional RTA structure, it allows contextual depth and complexity in understanding how hope and acceptance are intertwined, revealing how patients and clinicians fluctuate between hope and acceptance within a single consultation. While hope (e.g., hoping for hope) structure each section, these interactions naturally contain elements of acceptance, whether explicit or implicit. We therefore do not have a separate “acceptance” section.

### Hoping for hope

Freja, a woman in her late twenties, was in a traumatic accident about two years before the consultation. She has gone through extensive rehabilitation and most of her injuries are now healed. However, about one and a half years after the accident, Freja has started to experience a new pain. This pain is separate from the shoulder and neck pain still lingering after the accident, and it worries Freja. The consultation is not focused on the accident but on the various pain problems Freja is experiencing, which limit her ability to participate in physical activities and has led her to pause her education. Throughout the consultation Freja repeatedly express a wish for things to improve and a sense of uncertainty about her future:

Clinician: Freja, what do you think regarding expectations to coming here today?

Freja: Yes, no. I don’t know – no, of course I hope to get some hope maybe.

Clinician: Mhm.

Freja: In the sense that I would like to get better so that I can continue the education possibly next year. (.) And yes, get better. Be able to do the physical activities that I enjoy.”

She repeats this in the post-consultation interview:

TA: What expectations did you have for the consultation before you came?

Freja: No of course, something like that (.) yes as I said to [the clinician] that some hope that it will get better. That I won’t be like this for the rest of my life.

Freja does not say that she “expects” to get some hope, rather she emphasizes the potential unattainability by saying she “hope(s) to get some hope maybe”. Her ideal outcome from the consultation is not a diagnosis but hope for positive changes in the future. However, the clinician’s approach is largely centred around normalizing back pain and promoting acceptance:

You know that having back pain is very, very common. Almost eighty percent of the population will have back pain at some point in their lives and very often we don’t know what is causing the pain. (…) Ehm, maybe only for fifteen percent can we say that “we experience this as the reason for your back problems”.

Freja quietly affirms this—“mhm,” “yes” - but does not ask further. A little later, she tries to reorient the conversation towards possibilities for change:

Freja: What can I do with it for it to get better?

Clinician: Yes, for both things [pain problems]?

Freja: Yes.

Clinician: Then I think the best thing for you is to try to be in as much normal activity as possible. But you can’t just decide tomorrow – decide today that tomorrow you’re going to do the same thing you did before your accident.

(…)

Clinician: So, the best thing is if you can manage to just live as normal a life as possible uh but- and I’m not saying that tomorrow you’re going to go on a long ski trip or up in the mountains and so (.) but you have to build it up gradually. (.) and see how you respond. Yes. Uh and so uh so physical activity (.) is good for you, but I would also recommend continuing with yoga and muscle relaxation.

Freja: _mhm.

The clinician also refers Freja to a pain rehabilitation stay. But for Freja, these suggestions feel more like reminders of what she has already tried than a new direction. In the post-consultation interview, she reflects that little new came out of the encounter:

It was kind of very good, but the result of ((small laugh)) – the answer was not so much. Because I have – I already knew that I have to find a way to relax and not – yes kind of what I have tried with sauna and yoga and everything possible. So, there is not that much new information.

She describes the overall message of the consultation as:

Freja: (…) a bit like ((2)) “yes that’s how it is, everyone has back pain. We don’t know where it comes from, so you just have to accept it” and I think that is a bit (.) a bit like that (.) yeah.

TA: A bit weird?

Freja: Yes, a bit like “isn’t there something more we can do”.

TA: Mhm. So, were you missing a few more offers?

Freja: Yes.

TA: Yes.

Freja: Yes (.) a little more hope.

The normalization of the pain felt, to Freja, like a closing-down of possibilities, not a way forward. She does not reject reality—she knows this is hard—but the consultation does not offer her a future she can hold onto. The clinician’s nudge towards acceptance, though intended to reassure, unintentionally undermines Freja’s need for hope. Meanwhile, in the interview with the clinician, it becomes clear that Freja’s prognosis is actually fairly good:

It may well happen that Freja ends up having a neck and back that creates a bit of trouble and pain occasionally, but I think that she can learn nicely to live with it. Whether she will ever return to the level of function she had before the actual accident, I do not know for sure. (…) But there is a good prognosis.

The “good prognosis”, however, is not clearly communicated to Freja. In this sense, the consultation reveals a subtle but significant misalignment: between clinicians’ encouragement of acceptance and patients’ search and need for hope.

### Hoping for a quick fix

Jakob, who is in his late fifties, suffered a stroke three years prior to the consultation. He has since done rehabilitation to restore functioning but now experiences chronic pain in his leg and shoulder. Jakob has tried a variety of treatments to help with the pain, but nothing has worked so far. In the consultation the clinician identifies a potential cause of Jakob’s pain—lack of muscular control in one leg—and suggest that targeted physical training might be helpful:

Clinician: Because what I think moving forward is that it is wise for you to work on the problems you have with coordination, and some on pace and agility under the foot.

Jakob: Mhm

Clinician: Because it has so much to say about how you walk and stand and spasm and it can be one of the reasons for the pain you have.

Jakob: Mhm, but like why this feeling under the foot then – what does it come from?

Clinician: I’m a little unsure, it could come from what you had in your brain but (.)

Jakob: Yes

Clinician: But you say it started much later so it’s a little unlikely that it would be that.

Jakob:  < _yes

Clinician: but but your nerves seem to be working well.

Jakob: Mhm.

Clinician: But that it’s more the connection between the brain and the nerves that doesn’t work optimally.

Jakob: Okay.

Clinician: But regardless of what it stems from, then I think it’s a good idea to work on it.

Jakob: Yes yes, of course.

The clinician also reflects on Jakob’s previous pain treatments and highlights the difference between symptom relief and addressing underlying dysfunction:

Clinician: What I’m thinking, now you’ve tried many different measures on your muscles, both pressure waves and needle treatment and treatment from all sorts of uh specialist groups, but it hasn’t helped much.

Jakob: No it hasn’t.

Clinician: And I think that what they do is like putting out fires.

Jakob:  < yes.

Clinician: Because they treat your muscles but then you continue to walk like you do and climb stairs like you do and move really- maintaining it-

Jakob: Mhm.

Clinician: -By moving like you do. And then I think that it could be that this coordination and impaired balance and impaired feeling makes you move differently and use the muscles too much on one side and too little on the other side.

Jakob: Right.

Clinician: So working specifically with that and what is the cause of the problem and not just all the symptoms that come afterwards might be a good idea.

Jakob: Yes absolutely, I think so.

The clinician implies that improvement is possible with effort but stops short of explicitly stating the good prognosis. Although the clinician communicates a belief that targeting these coordination issues may help, this is presented in a cautious and qualified manner. This in contrast to the clinician’s reflections in the post-consultation interview:

I hope that Jakob realises that these are things that you can work on getting better. But that he has to make an effort himself. (.) um with physical um training, and and effort, then it can get better. (…) it is not every time we can give concrete treatment suggestions and that we actually think that ‘this is something different than what you have done earlier and maybe it will work’.

Clinicians rarely offer such concrete treatment suggestions and express hope for improvement. However, the clinicians reserved phrasing during the consultation might have affected how it was received, as Jakob does not appear to have fully understood the positive potential of the findings:

TA: To what extent did the consultation give you more clarification in relation to your condition?

Jakob: Uh, there’s probably no clarification in that way about uh- other than that I have to contact (.) a manual therapist, and maybe what kind of exercises I should focus on doing myself at home.

TA: Yes.

Jakob: So uh (.) it probably won’t work, you have to try.

TA: Mhm. (.) So, what is your perspective on the future?

Jakob: Uhm, one has to be an optimist ((laughter)) and hope that things will get better.

Jakob’s reflections are cautious and tinged with doubt, mirroring the ambivalence seen in his expectations and hope for the consultation:

No, well, I understand that it’s difficult to find out, like “what is the cause” and “where does it come from”, so I didn’t expect that there would be a quick fix for it. I didn’t expect that. But uhm, maybe to try to find out a little more and get a step further towards (.) getting rid of the problem.(…)I had hoped that uh they would be able to say that uh “then we have to try to do such and such because the cause is this ligament here”. I do – I understand that it is of course difficult to say that uh (.) that it is specifically “that” which is the cause of it. That I walk wrong or something ((laughter)).

Jakob draws a distinction between his expectations, which do not include a “quick fix” or diagnosis, and his hopes which longs for one. Although this case involved a relatively rare scenario where the clinician could offer a potential explanation for some of the pain and a targeted treatment path, the communication did not seem to instil hope. Jakob’s doubt may stem from past extensive experiences with various types of therapy (including physiotherapy) that yielded little benefit. However, it may also reflect the clinician’s reserved way of expressing optimism. Interestingly, Jakob’s hope—that someone would tell him “you’re walking wrong or something” - is quite close to what the clinician suggested during the consultation. Yet Jakob did not seem to recognize this as the kind of explanation he had hoped for. As such, the opportunity to convey the potential benefit of physical therapy—which this case arguably held—was not fully realized.

### Hoping for a diagnosis

Anne, a woman in her early thirties, has recently received a rheumatic arthritis (RA) diagnosis for pain presenting in her hands and feet. The treatment has removed this pain, but new pain not attributed to the RA diagnosis—located in her shoulder and low back—has emerged and worsened over time. Uncertain of its origin, her rheumatologist referred her to the pain clinic. Yet, when Anne met with the clinician there, it became clear that neither party had a precise understanding of the referral’s purpose.

Clinician: Uhm so what do you want to get out of the referral here?

Anne: She [rheumatologist] talked about sending me to, um, that kind of rehabilitation stay.

Clinician: Yes. Is that something you would like?

Anne: I don’t know what I would like. What I would like is to get rid of the pain in my back – now it’s in my back. I had almost hoped that I would have such Bekhterev’s [ankylosing spondylitis] when I got arthritis. And when I was diagnosed with arthritis, they told me that the pain I had in my back could be due to arthritis and that when I started medicine it could get better. But it was only the joints that got better, it didn’t get better in the back.

(…)

Anne: At least that’s how it felt (.) and now I have tried- I started training with a physiotherapist in January.

Clinician: Mhm.

Anne: And on my own and I think it just triggers the pain more.

Clinician: Yes.

Anne: And it’s not getting better and I don’t know what to do about it.

Clinician: No. (.) So (.) if I understand you correctly then- mostly now you’re suffering from back pain and hoping to get some help with that.

Anne: Yes.

Clinician: Uh they had talked about a rehabilitation stay, but you don’t really know what you’re thinking.

From the outset, Anne reorients the conversation away from general rehabilitation towards a hope for diagnostic clarity or therapeutic intervention. Having a clinical diagnosis like Bekhterev would provide her with answers and possible ways to improve her condition and she “really just hoped that there was something wrong, that it could be operated on or that it was so easy (…)”. In the post-consultation interview, Anne describes how relieved she was when getting the RA diagnosis because “there was actually something wrong. You start to wonder if you are a hypochondriac”. For Anne, diagnosis carries more than clinical utility—it provides legitimacy. It means there is something real to hold onto, something not imagined, not dismissed. The medical name is the starting point for defining, explaining, and acting on our illnesses, and for predicting future developments. Nameless ailments lack this fundamental starting point, and thereby remain indecipherable((Nettleton, 2006).

As the consultation progresses, the clinician begins to steer away from the possibility of any new diagnosis. Instead, the conversation shifts to how Anne might live with ongoing pain—towards a narrative of acceptance, adjustment, and self-management.

Clinician: If you think you have a very good job and you don’t want to re-educate. (.) So if you can’t do everything at the moment then maybe you simply have to cut back a little. ((pause 2 sec)) and acc- try to accept (.) now you have rheumatism and you have to take care of it.

Anne: Yes, I think it’s rather that I have to learn.

Clinician: Mhm.

Anne: _That _I _am _actually _sick _and _cannot _keep _up _with _so much.

While Anne nods to this pragmatic turn, her agreement appears to come more from resignation than conviction. In the post-consultation interview, she reflects:

I am not really a negative person, but when it comes to my back, I am a bit negative because I have been to so many people and have been bothered for so long that I have really lost hope.

Anne has previously experienced not being believed by clinicians or being promised improvements that did not happen. This experience now manifests as a slight mistrust when clinicians say that her back pain is “nothing serious”. While her hope for improvement and a diagnosis is not easily given up, she simultaneously feel hopeless. When Anne is told her pain is likely muscular, the news lands with quiet disappointment “But [I] only had a hope that you ((2)) that it should be something else.”. Unlike RA or Bekhterev’s, Muscular pain lacks diagnostic weight; it does not authorize intervention or carry a clear treatment path and Anne wants specificity:

Anne: I just feel like I need to know exactly what is wrong with my back and know exactly what I should do ((small laughter)).

Clinician: Yes (2) it is not sure that there’s anything exact.

Anne: No but something that helps at least.

Knowing that it is muscular therefore does not give Anne hope that anything will change. While the clinician explain how chronic pain can become entrenched and how physiotherapy, training, and relaxation techniques could help, Anne feels she is already doing what is asked: “Yes but I am doing all of that all the time”. She feels like she is pushing through, maintaining balance, self-regulating, yet the pain remains and while she got answers from the consultation, she remains caught on the idea that it might be something that the clinician has not found like Bektherevs:

Because in the beginning when I had trouble with my foot, they thought it was because I was putting the used my feet wrong. (…) So, I didn’t trust them. (…) Then after many years they found out it was arthritis. So, I thought that this (.) this shouldn’t last multiple years.

Without a clear diagnosis, Anne is left navigating the uneasy space between holding on to hope and learning to live with what cannot be explained.

### Hoping for surgery

Emil, in his early fifties, presents with neck, hip, and back pain. Although he was in an accident nine months ago, the pain began six months later—making a direct link uncertain. He was initially assessed for spinal surgery, but with no clear indications, was referred to the pain clinic instead. Emil opens the consultation with hope for clarity and resolution:

Clinician: Yes, welcome here.

Emil: Yes, thank you, I hope you can make me a bit wiser with my questions.

Clinician: A little wiser?

Emil: Yes.

Clinician: Yes, because that you need to become …

Emil: Yes, I hope I can figure out these problems of mine, because it’s not very nice to go around like that, soo…

In the post-consultation interview, Emil also admits he had hoped for a surgical re-evaluation:

(…) I didn’t really know what I was going into. I had actually thought that maybe I should uh talk about maybe taking new pictures to see if there was a change or if uh if it was something that was possible to operate on (…). When I was with the GP last time and she referred me, I might have had a little hope of surgery.

The clinician anticipated Emil’s focus on surgery but had other expectations, as discussed in the interview:

I expect that it is an uh active man with pain in the neck and back of which he has some expectations will be operated on and he will get better. Uhm so it is a bit of exploring and then see if we can come to an agreement on some conservative measures.

Emil and the clinician thus enter the consultation with misaligned goals and expectations: Emil hopes for surgery; the clinician aims for conservative management.

Throughout the consultation, Emil voices frustration with the lack of answers and treatment and compares himself to a friend who improved after surgery—fuelling his hope for similar treatment. The clinician, meanwhile, emphasizes a more holistic view of the body—“the body which is here now, today” - and concludes, following a physical examination, that surgery is not needed: “From an examination perspective, I find no nerve compression in the neck or back. That’s very positive,”. The clinician proposes lifestyle changes and rethinking Emil’s work:

Clinician: Mhm (.) have you started – have you also started to think together [with GP] about whether you should try something else [workwise] that is not so sedentary _like _like-

Emil: yes, that is indeed kind of like the thing, just like what it should be. That uhh – I just feel, like I say with that – when I have to find out what to do when I have to exercise.

(…)

Clinician: What’s important is to find activities you can manage and feel okay about. Find the right dose of exercise — how long can you keep going? Can you do shorter sessions? There’s also a five-day program at the rehabilitation center, with a focus on coping and work, that I think might be relevant for you. If you want, I can refer you.

It becomes increasingly clear that for Emil this “positive” result is opposite of his desired outcome, and he is not yet ready to accept pain as a chronic condition. He focuses on “doing something” that might fix the issue. The clinician tries to offer tangible alternatives, but Emil seems stuck on surgery, possibly because he had pinned significant hope on this consultation. Although the clinician finds no clinical signs justifying surgical intervention, they try to acknowledge Emil’s ongoing uncertainty and unmet expectations. Rather than closing off the surgical option completely, the clinician offers to take his case back to the neurosurgical team:

Clinician: So, since you’re talking about surgery, I can take this to the neurosurgeons and hear what they think — especially regarding those other surgeries that have been considered. I don’t think surgery will happen here, but they might have thoughts about the disc [substitute] or other options elsewhere in Norway.

Emil: Yeah, because I feel like I haven’t gotten any wiser today, really. I’ll be going home with an unchanged case.

Clinician: Yes. I think I’ve gotten as far as I can today in terms of the examination. These are the findings. Now it’s more about the next steps — what kinds of interventions we can try.

The clinician later reflects that Emil “persuaded” them to take the case further with the neurosurgical team:

Clinician: But Emil now persuaded me to take it further.

TA: Mhm.

Clinician: So ((2)) but of course everything is not black and white all the time. Some are very ready to go ahead and have surgery and some do have the [good] effect of surgery. So, to completely remove Emil’s hope it was wrong of me to do that. So now you can take it further and then you can see what the possibilities are and if he wants a further referral for a second opinion then I think that then I can send him somewhere else if he wants it.

The clinician thus balances clinical judgement with a desire not to crush Emil’s hope outright. To do so the clinician perceived as wrong. However, sustaining this hope by passing the decision on to another clinician risks merely postponing the disappointment. In fact, by agreeing to further referral, the clinician may unintentionally reinforce Emil’s belief that surgery is still a viable—and perhaps likely—option, increasing his hope, perhaps only for it to be taken away again.

At the very end, the clinician tries to bridge the gap between Emil’s hopes and the medical realities by also bringing up the emotional strain Emil is experiencing and suggests another form of support:

Clinician: And I hear you say that this really affects your mood — that you’re feeling down?

Emil: Yeah, it makes you depressed. You feel sad, because you want to do the things your friends do, and you can’t.

Clinician: Would you like to look into that further? I can set you up with a psychologist to work on some tools for that. What do you think — would you be open to an assessment?

Emil: Yeah, sure. I’ll try anything.

The clinician was positively surprised that Emil was willing to take this further, however, in the post-consultation interview, Emil emphasizes that being reconsidered for surgery was the most meaningful part of the visit. Without the potential of surgery, Emil would have felt like he left the consultation with nothing. In this sense, the clinician’s decision to sustain his hope, left Emil with a positive experience where he otherwise would have been dissatisfied. The cost of preserving hope in this way is that it may delay rather than facilitate acceptance.

## Discussion

Clinical encounters with CMP patients are fraught with challenges surrounding expectations, hope, and acceptance. While patients were unsure what to expect, many held onto hope for biomedical explanations and solutions as a diagnosis or treatment. In contrast, clinicians worked to promote acceptance and reassure, supporting symptom management rather than continuing to seek a cure. By shifting the focus from explanation to acceptance, clinicians reframed the goal of the consultation. However, patients’ expectations and hopes, might mean they are not yet ready to accept the pain or finds acceptance dispiriting. This tension creates a complex dynamic in the consultation room.

### Acceptance without hope

Our findings align with research showing that clinicians must carefully balance hope while managing expectations (Hagerty et al., 2005; Kube, Blease, et al., 2019). Because hope in itself contains uncertainty, it is not necessarily lost if disappointed, but as our data show pushing acceptance might undermine hope and create resistance. When nudged towards acceptance, it can thus result in the patient feeling hopelessness and disbelief as Freja did.

It is generally acknowledged that having hope is essential (Lohne, 2022) but because of the uncertainty linked to chronic pain, patients like the ones in our study, continuously balance between hope and despair (Eaves et al., 2016). Clinicians in our study rarely stated explicitly that the prognosis was good, even when they aimed to reassure patients. This may reflect a desire to avoid false hope, but the lack of a clear positive outlook can deprive patients hopes for future improvements. Freja’s desire to leave the consultation with hope was disrupted when acceptance was framed as final, especially as the reassuring outlook was never explicitly communicated.

The word “acceptance” may carry different meanings. Clinicians see it as an empowering step towards coping (McCracken et al., 2004), while patients might interpret it as and think they have to resign themselves to *all* experiences of suffering (McCracken et al., 2004). For patient, hope and acceptance can thus come to be perceived as opposites, where hope represents an open (yet uncertain) future and acceptance represents a closed (and to a higher degree certain) future. The challenge with this understanding is that it might be virtually impossible to accept status quo without any hope at all. Just as hope indicates an open future, acceptance must also, to a certain extent, refer to an open future in order to be bearable. Acceptance might therefore not be attainable without hope. Hope as such plays a crucial role in pain acceptance and maintaining hope without giving “false” hope is a nuanced skill that requires careful balancing.

One study found that people living well with chronic pain have accepted the pain as part of their life and engaged in a process of re-occupying self to establish self-coherence (Lennox Thompson et al., 2020, p. 1147). Part of this process is what the authors refer to as “sense-making”. This includes naming the disease in order for the patient to move on rather than searching for a diagnosis (Lennox Thompson et al., 2020, p. 1147). In If acceptance is reliant on naming the condition then this process is further complicated in CMP consultation where a diagnosis is absent.Kornelsen et al. (2016, p. 374) have suggested, that instead of leading to despair and isolation, the clinician’s inability to provide a diagnosis or cure should lead to a shared intimacy and acceptance of uncertainty (Kornelsen et al., 2016). However, in the literature there is little theoretical or practical guidance on how this should be done and uncertainties in general should be navigated.

In their study, Lannie and Peelo-Kilroe (2019) found that cancer patients’ acceptance was commonly linked to patients’ age. Framing acceptance through age and life experiences, older cancer patients were more prepared to accept their illness, while still maintain hope. However, their hopes took on different characteristics, like spending time with family or tending their garden (Lannie & Peelo-Kilroe, 2019). The fact that hope changes with time has been documented in the chronic pain literature. Patients shift from hoping for a speedy recovery to focusing on self-management over time (Eaves et al., 2015; Lohne, 2022). Patients in our study may not yet have been at a stage where they were ready to accept the pain, but that they might get there with time as their hope changes. In settings like therapy, patients may be guided over time to develop these coping mechanisms, but in singular consultations like the ones in this study, there are limits to how far clinicians can guide patients towards acceptance in one encounter. However, removing patients current hope by pushing acceptance, might leave them unable to uphold the equilibrium between hope and despair, leaving them hopeless. It is therefore important for clinicians to understand where patients are on this “timeline” and adapt their strategies accordingly.

### The weight of cultural expectations to biomedicine

The clinical consultation is situated in a contemporary biomedical health system which, to a large degree, is based on a problem-solution format and a technology-dominated biomedical perspective (Nettleton, 2013; Reiser, 1978). Furthermore, the wider sociocultural surroundings of Western Europe carry high expectations of—and faith in—the medical professionals’ knowledge and skills, resulting in strong expectations that biomedicine can provide accurate diagnoses, identify causes of illnesses, and offer effective treatments (Brekke & Sirnes, 2011; Freidson, 1970). Despite this reliance on biomedical solutions, acceptance and self-management strategies are sometimes the only treatment clinicians can offer, however this is often not what patients hope for.

In interviews, patients expressed confusion about the purpose and potential outcome of the consultation. In most cases, the patient knew that s/he was going to a specialist clinic for a consultation about long standing symptoms, and that the clinicians might know more. Without more clarity, the patients could not estimate the probability of a certain outcome, and they orientated to the uncertainty by reframing their answer as a hope. Studies have shown that patients’ estimation of probability and desirability of a particular outcome, is based on their own and others’ prior knowledge and experiences (Leung et al., 2009, p. 357; Thompson & Suñol, 1995). The patients in our study have been suffering from musculoskeletal pain for a while, and as such they have seen many practitioners and have been disappointed repeatedly when no answers or solutions could be found. Their expectations for the consultation and the future were low because of these previous experiences with the health care system as explained by Anne (“ … I have been to so many people and have been bothered for so long that I have really lost hope”).

This is similar to other studies that show patients counterbalance hope by keeping low expectations to treatments (Eaves et al., 2014). However, while expectations are typically fixed and limited to our (and others) previous experiences, hope is often flexible and not limited by experiences. It acknowledges reality but does not resign itself to it. Because of this, the patients hope, despite themselves, that this might be the consultation where the clinician can tell them what is wrong or at least how to get better. In this way the uncertainty of the clinical encounter and the surrounding cultural faith in biomedicine and possible scientific breakthroughs (Brekke & Sirnes, 2011; Sulmasy et al., 2010) allows patients to hold onto hope amid low expectations. The patients are thus willing to hope that they might get a diagnosis or a “quick fix” but reluctant when it comes to believing that self-managed treatment might alleviate their pain. The difference between the two, lies with the source of the solution. A diagnosis or “quick fix” is a medical solution which does not require much involvement from the patient, whereas self-managed treatment such as exercise, is fully reliant on the patient.

In our data, Emil explicitly articulates his hope that medicine is able to provide a solution to his problem. The patient’s belief in medicine and search for a biomedical solution in conflict with the clinician’s more holistic approach, is contrary to what we have previously seen in literature. Often the clinician’s biomedical perspective is juxtaposed to the patient’s bodily experience (see for example Lannie & Peelo-Kilroe, 2019). However, here Emil is the one adhering to the biomedical perspective. This shows how patients carry the weight of the cultural expectations to biomedicine into the consultation, wanting biomedical explanations and solutions for their problems. Hence, while attempting to adjust their own biomedical perspective and employ a more holistic approach, clinicians also have to navigate the cultural image of biomedicine as the solution persistent in society and present in the patient.

That the clinician admits that Emil persuaded her/him to go further with the neurosurgeons, despite believing this will not lead to anything, indicates that clinicians are under pressure. To avoid removing Emil’s hope, the clinician agrees to take his case further, prolonging the hope and leaving it to someone else to disappoint him. The clinician does not believe it is her/his role to take hope away from the patient. However, it could be argued that it is exactly the clinician’s responsibility to balance this hope, avoiding encouraging the patient only for that hope to be taken away later.

Through Freja’s, Jakob’s, Anne’s, and Emil’s story we have shown just how entangled the concepts of expectations, hope and acceptance are in the clinical encounter and how difficult they can be to navigate for both parties. Instilling hope in patients is a powerful skill and one that clinicians need to balance so that they neither exaggerate nor take away hope. These findings highlight the need for clinician education that strengthens communication skills around managing hope and guiding acceptance. Recognizing where patients are in their illness journey—and adjusting messages accordingly—may help clinicians avoid undermining hope while still promoting realistic expectations. This involves small but meaningful shifts in how acceptance is introduced and framed, emphasizing that acceptance does not mean giving up, and that patients are not alone in navigating this process.

## Strength and limitations

While this study did not originally set out to explore expectations, hope and acceptance in clinical encounters, these concepts emerged through our analysis of the data. The study’s strength lies in its combination of observational and interview data which provided comprehensive insights into how patients and clinicians perceived the interaction while being able to compare it with what actually happened in the consultations. We acknowledge that participants might change their behaviour, knowingly or unknowingly, because they are observed, and their talk recorded. However, observations allowed us to witness first-hand the dynamics and nuances of the clinical encounters, capturing non-verbal communication, behaviours, and contextual factors that may not be fully conveyed through interviews. The interviews, on the other hand, offered a deep insight into patients’ and clinicians’ experiences, thoughts, and feelings. While interviews took place in the clinic, which might have influenced the participants stance, they also provided context and meaning to the observed behaviours and interactions, offering a more complete picture of the clinical encounter. Excluding patients with language barriers may have limited the diversity of communicative experiences and affects generalizability. We acknowledge that our findings represent only certain types of clinical encounters, however the basic interactional elements might occur beyond the study group.

## Conclusion

Hope and acceptance are different, but closely related, responses to the central experience of uncertainty in chronic musculoskeletal pain consultations. Patients enter consultations equipped with Western European cultural expectations of biomedicine, seeking biomedical explanations and solutions for their problems. They orient to the inherent uncertainty of CMP, by reframing their expectations as hope, often basing this on their faith in biomedicine. This study illuminates a challenging balancing act in these clinical interactions; patients having realistic expectations of future developments and accepting their condition, while at the same time maintaining their hope and strive for improvements. This dual task of maintaining hope and attaining acceptance, both of which are crucial, can be challenging to carry out. This is because hope and acceptance might be seen as opposites by the patient, in the sense that hope represents an open future and acceptance a closed one. We found that the patients were not yet in a stage of their illness journey where their hope could align with acceptance. In this case, for acceptance to be attainable and bearable it must also imply an open future and thereby a sense of hope.

These findings highlight the need for clinical training to help practitioners recognize patients’ illness stages and communicate acceptance while also sustaining their hope for a better future. Emphasizing positive prognosis and the possibility of living a more pain-free life might support this balance. Improving clinician communication skills is essential to navigate these challenges effectively.

## Data Availability

The participants of this study did not give written consent for their data to be shared publicly, so due to the sensitive nature of the research supporting data is not available.
